# Supplementary material for: Systematic Review of Studies on Telomere Length in Patients with Multiple Sclerosis
Source: Aging Dis. 2021 Aug 1;12(5):1272–86. doi: 10.14336/AD.2021.0106 (PMC8279528; doi:10.14336/AD.2021.0106)
Supplement: Supplementary file 1 [file AD-12-5-1272-s.pdf]

SUPPLEMENTARY DATA

Systematic Review of Studies on Telomere Length in Patients with Multiple Sclerosis

Jan Bühring[#], Michael Hecker[#,*], Brit Fitzner, Uwe Klaus Zettl

© 2021. Bühring J et al. Published online at http://www.aginganddisease.org/EN/10.14336/AD.2021.0106

SUPPLEMENTARY DATA

Supplementary Table 1. Full list of studies assessed for inclusion in this systematic review.

Number	First author	Year of publication	PubMed or preprint identifier	English or German?	Original article?	Samples from patients with MS?	Measurement of TL?	Inclusion in review
1	Olerup	1990	1978948	yes	yes	yes	no	-
2	Charmley	1991	1674514	yes	yes	yes	no	-
3	Spurkland	1994	8071104	yes	yes	yes	no	-
4	Roth	1995	7499175	yes	yes	no	no	-
5	Torelli	1995	7561787	yes	yes	yes	no	-
6	Pham-Dinh	1995	7590972	yes	yes	no	no	-
7	Roth	1995	7593547	yes	yes	yes	no	-
8	Encinas	1996	8757345	yes	yes	no	no	-
9	De Boer	1998	9637494	yes	yes	no	no	-
10	Morris	1999	10223549	yes	yes	no	no	-
11	Bihl	1999	10224268	yes	yes	no	no	-
12	Allcock	1999	10369924	yes	yes	no	no	-
13	Stefferl	1999	10384097	yes	yes	no	no	-
14	Kalman	1999	10554670	yes	no	no	no	-
15	Allcock	1999	10626741	yes	yes	yes	no	-
16	Oksenberg	2000	11164900	yes	no	no	no	-
17	Encinas	2001	11222494	yes	yes	no	no	-
18	Jawaheer	2001	11254450	yes	yes	no	no	-
19	Marrosu	2001	11741834	yes	yes	yes	no	-
20	Storch	2002	12146797	yes	yes	no	no	-
21	Mellai	2003	12559630	yes	yes	yes	no	-
22	Hug	2003	12817027	yes	yes	yes	yes	✔
23	Duvefelt	2003	14651518	yes	yes	yes	no	-
24	Rubio	2004	15014978	yes	yes	yes	no	-
25	Jagodic	2005	15634914	yes	yes	no	no	-
26	Muhallab	2005	15748954	yes	yes	no	no	-
27	Vyskhina	2005	16078049	yes	yes	yes	no	-
28	Weksler	2005	16141364	yes	yes	no	no	-
29	Thewissen	2005	16154497	yes	yes	yes	no	-
30	Marrosu	2006	16096810	yes	yes	yes	no	-
31	Saarela	2006	16596167	yes	yes	yes	no	-
32	Lamoury	2006	16793732	yes	yes	no	no	-
33	Rubio	2007	17256150	yes	yes	yes	no	-
34	Cailier	2008	18832704	yes	yes	yes	no	-
35	Ordóñez	2009	19421224	yes	yes	yes	no	-
36	Baecher-Allan	2011	21300823	yes	yes	yes	no	-
37	Montoya	2012	22770640	yes	yes	no	no	-
38	Oksenberg	2013	24289837	yes	no	no	no	-
39	Westerlind	2015	25159868	yes	yes	yes	no	-
40	Guan	2015	25424527	yes	yes	yes	yes	✔
41	Reddy	2017	28253983	yes	no	no	no	-
42	Cebrián-Silla	2017	28648897	yes	yes	no	no	-
43	Redondo	2018	28548004	yes	yes	yes	yes	✔
44	Guan	2018	30092167	yes	yes	yes	yes	✔
45	Liu	2019	31294790	yes	yes	yes	no	-
46	Din	2019	31407831	yes	yes	yes	no	-
47	Krysko	2019	31486104	yes	yes	yes	yes	✔
48	Habib	2020	32050150	yes	yes	yes	yes	✔
49	Carraro	2020	32499887	yes	no	no	no	-
50	Liu	2020	bioRxiv 2019.12.19.882522	yes	yes	no	no	-
51	Hecker	2020	medRxiv 2020.11.17.20232975	yes	yes	yes	yes	✔

The literature search was conducted using the databases PubMed, bioRxiv and medRxiv with the search terms "multiple sclerosis" and "telomer*". This resulted in 51 articles that were screened and assessed for eligibility (sorted by year of publication). Based on previously defined study selection criteria, we excluded 44 of these articles. The included studies (marked by ✔) were original research articles written in English or German that presented data on telomere lengths (TL) measured in samples from patients with multiple sclerosis (MS).

© 2021. Bühring J et al. Published online at http://www.aginganddisease.org/EN/10.14336/AD.2021.0106
